# Adaptive temporal compression for reduction of computational complexity in human behavior recognition

**DOI:** 10.1038/s41598-024-61286-x

**Published:** 2024-05-08

**Authors:** Haixin Huang, Yuyao Wang, Mingqi Cai, Ruipeng Wang, Feng Wen, Xiaojie Hu

**Affiliations:** 1 School of Automation and Electrical Engineering, Shenyang Ligong University, Shenyang, 110159 China; 2 School of Information Science and Engineering, Shenyang Ligong University, Shenyang, 110159 China

**Keywords:** Human behavior recognition, Video analysis, 3D convolution, Adaptive, Compression technology, Computational science, Computer science, Information technology

## Abstract

The research on video analytics especially in the area of human behavior recognition has become increasingly popular recently. It is widely applied in virtual reality, video surveillance, and video retrieval. With the advancement of deep learning algorithms and computer hardware, the conventional two-dimensional convolution technique for training video models has been replaced by three-dimensional convolution, which enables the extraction of spatio-temporal features. Specifically, the use of 3D convolution in human behavior recognition has been the subject of growing interest. However, the increased dimensionality has led to challenges such as the dramatic increase in the number of parameters, increased time complexity, and a strong dependence on GPUs for effective spatio-temporal feature extraction. The training speed can be considerably slow without the support of powerful GPU hardware. To address these issues, this study proposes an Adaptive Time Compression (ATC) module. Functioning as an independent component, ATC can be seamlessly integrated into existing architectures and achieves data compression by eliminating redundant frames within video data. The ATC module effectively reduces GPU computing load and time complexity with negligible loss of accuracy, thereby facilitating real-time human behavior recognition.

## Introduction

**Figure 1:**
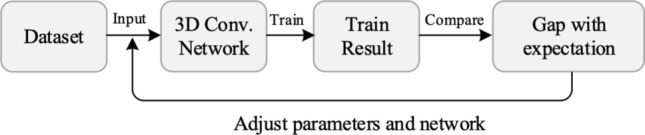
3D convolution for human action recognition.

Human behavior recognition is a significant research area in computer vision. The traditional 2D convolutional feature extraction from videos has several limitations^1–3^. For instance, the neglect of temporal information can result in poor feature capture and classification errors for neural networks. In contrast, 3D convolution has the ability to extract spatio-temporal features accurately and capture temporal flow information, thereby significantly improving the neural network’s capacity for human behavior recognition^4,5^. The process of training a deep convolutional network for human behavior recognition involves the initial input of the dataset, followed by recording the training results such as loss and accuracy, see Fig. 1. Subsequently, error is calculated and backpropagation is performed to adjust the network parameters in order to enhance the model performance.

However, as video datasets continue to expand and the increase of parameters of 3D convolution, it leads to the rise in time complexity^6–8^ and a greater dependence on GPU hardware for model training. Hence, this accelerates the research of innovative approaches for human behavior recognition through the use of deep learning algorithms and hardware optimization.

Human behavior recognition technology heavily relies on videos as inputs, which generate a large number of frames for each type of action and corresponding video clip. For instance, datasets such as UCF101^9^ and Kinetics^10–13^ can range from tens to hundreds of gigabytes. In addition, deep learning networks based on 3D convolution, such as C3D^4^, I3D^10^ and S3D^14^, require spatial and temporal feature extraction for all frames, resulting in extensive computation of image matrices. However, due to the high computational demands, only a few laboratories with powerful GPU and parallel computing capabilities can achieve optimal training speeds. As a result, the vast majority of researchers are unable to afford high-performance GPU training environments, which severely restricts experimental efficiency. Novel approaches that address these computational challenges and increase the accessibility of high-performance computing environments are therefore critical to the development of efficient human behavior recognition technology.

In the field of behavior videos analysis, the significant increase in the number of parameters and high computational complexity pose significant challenges. The main contributions of this study are as follows:

- The ATC module is a seamlessly integrable module. It is capable of compressing datasets by removing redundant video frames with minimal loss of relevant information.
- The ATC module reduces the number of training and testing samples by compressing data, thereby lowering the computational load and time complexity of the model.
- Experimental results demonstrate that this approach enhances experimental efficiency and model performance with negligible loss of accuracy.

## Related work

Due to the outdated hardware equipment and the lack of effective extraction of video data features in the early days, traditional behavior recognition relied mainly on manual feature extraction to establish corresponding behavior models, which were then utilized to complete behavior recognition tasks^15,16^. The holistic representation method identifies human behavior in image sequences by extracting geometric features and motion information and represents actions through the 3D shape in the spatio-temporal domain by encoding relevant motion information in the image. Researchers have also explored local features such as scale-invariant feature transform (SIFT)^17^ points or spatio-temporal interest points (STIP)^18^ in the spatial domain to describe action information without correlation. Compared to the holistic representation method for behavior recognition, this approach can more effectively capture behavior characteristics and reduce the impact of occlusion.

With the rapid development of deep learning and computer hardware, applying it to video analysis has addressed the problem of insufficient accuracy in manual feature representation and avoided the subjectivity and variability in the process of designing features manually. To capture a connectivity between static images and dynamic processes, Simonyan et al.^19^ proposed the Two-Stream network, which calculates dense optical flow for every two frames of the video sequence and uses the video image and dense optical flow as inputs to two independent networks. However, this approach is not entirely end-to-end video analysis, as it requires offline computation of optical flow and cannot achieve real-time processing.

According to Donahue et al.^20^, the key to video analysis is learning temporal features. Therefore, they proposed the fusion (CNN-LSTM) structure by combining CNN with LSTM to extract spatio-temporal information from video data. Other researchers have also combined the GCN network with human skeletal features, such as Yan S et al. put forward ST-GCN network^21^, which uses graph convolution to extract skeletal spatial features and time convolution to obtain temporal features, and then fuses the two for experimental results. C3D (3-Dimensional Convolution) action recognition is also a major method^4,22,23^. This method is much faster than the Two-Stream method, and is mostly trained end-to-end with a simpler network structure. Tran et al.^24^ constructed a network using 3D convolution and pooling that can directly process videos (or video frame volumes) and extract features for video-based problems.

However, training end-to-end networks requires significant computational resources and may result in overfitting and data redundancy due to a high number of parameters. Almost all CNN networks struggle to run on resource-limited systems. Therefore, tackling the issues of the explosive growth of 3D convolutional parameters and slow training is of utmost importance. Han et al.^25^ utilized weight sparsity through a combination of pruning, quantization, and Huffman coding to compress network structures. Srinivas et al.^26^ applied sparse constraints to each weight by using additional gate variables and pruning links with zero gate values to achieve high compression rates. Most existing 3D convolution methods optimize the network at the layer level^27–29^, training and testing the entire dataset as input, ignoring dataset-level issues. Considering that adjacent video frames may be highly similar (redundant) after video frame extraction, removing redundant video frames can reduce training time, improve experimental efficiency, and enhance model performance.

Several scholars have made significant efforts to explore keyframe extraction methods that can convert video processing into image processing. For example, Gharbi^30^ and colleagues proposed a keyframe extraction method based on local description and graph modular clustering. Guan^31^ and colleagues proposed a keyframe selection method based on keypoints, which can detect the differences in similarity between consecutive frames, but may extract similar keyframes and encounter issues such as a drastic increase in computational complexity or ignoring valid information. The human visual system can recognize and construct incoherent videos. According to research, video representation learning is accomplished by predicting the positions and durations of incoherence in order to maximize mutual information and learn advanced representations^32–35^.

In order to reduce the correlation between extracted key frames in video, Sunkara et al.^36^ proposed using the SPIHT (Set Partitioning in Hierarchical Trees) algorithm, which uses wavelet transform to convert the various groups of images captured from the video into one or several images with high spatial correlation. This method can effectively compress videos, and has a significant effect in high bit rate and slow-motion videos. On the other hand, Waingankar et al.^37^ employed Discrete Cosine Transform (DCT) to decorrelate the images, and further reduced the video signal data using optimized Huffman coding, achieving a compression efficiency of up to 85% with Peak Signal to Noise Ratio (PSNR) over 40DB.

To reduce the computational resources and time complexity required for video data analysis, an Adaptive Temporal Compression (ATC) module is proposed in this study. ATC is a seamlessly integrable module capable of efficiently identifying redundant frames within datasets and removing them without affecting the existing architecture. Unlike other video compression methods, ATC integrates the remaining video frames and utilizes them as compressed datasets for deep learning network utilization.

## Methods

In this section, we will first review popular 3D convolutional networks for human action recognition, and then provide a detailed introduction to the integration process, working flow, and functionality of the ATC module with the network.

### 3D convolutional network

**Figure 2:**
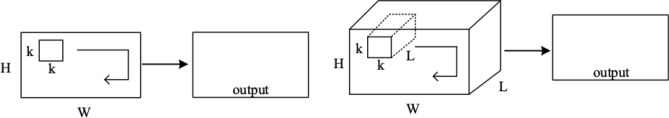
2D convolution operations.

The traditional 2D convolutional network is designed to extract features from individual images. It is unable to capture information along the temporal axis. As result, the network produces an independent feature map, as shown in Fig. 2. The $(H\times W)$ size picture or the $(H\times W \times L)$ size video is subjected to two-dimensional convolution with a $(k \times k)$ size convolution kernel, and the output is an independent feature map. Because it fails to capture the temporal information, this type of network is not ideal for tasks that require the analysis of video sequences, such as human behavior recognition.

**Figure 3:**
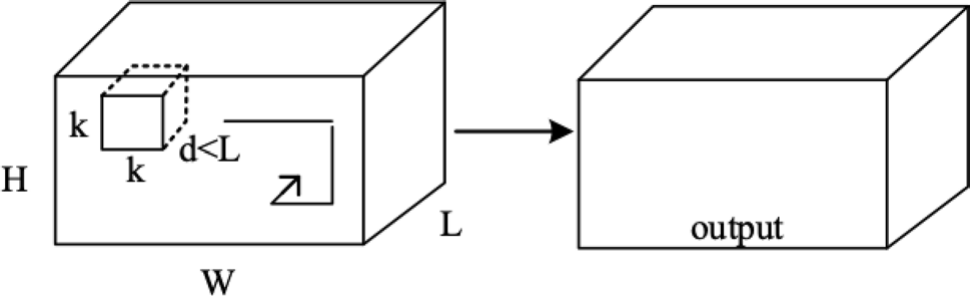
3D convolution operations.

The 3D convolutional network is capable of extracting spatio-temporal information from video frames, which enables it to analyze and predict input videos more accurately than the 2D convolutional network, see Fig. 3. This is because the 3D convolutional network can extract temporal information while maintaining the accuracy and efficiency of spatial feature extraction. When applying a 3D convolution for processing a video of dimensions $(H\times W \times L)$, the $(k \times k)$ two-dimensional convolution kernel is transformed into a $(k \times k \times d)$ three-dimensional form. The output is a cube comprising dependent multi-frame correlation feature maps that encompass characteristic information of both time and space dimensions. Specifically, the spatio-temporal information extracted by the 3D convolutional network is utilized to classify human behavior, such as swimming, archery, skateboarding, crawling, and yoyoing, among others, in the context of human behavior recognition.

**Figure 4:**
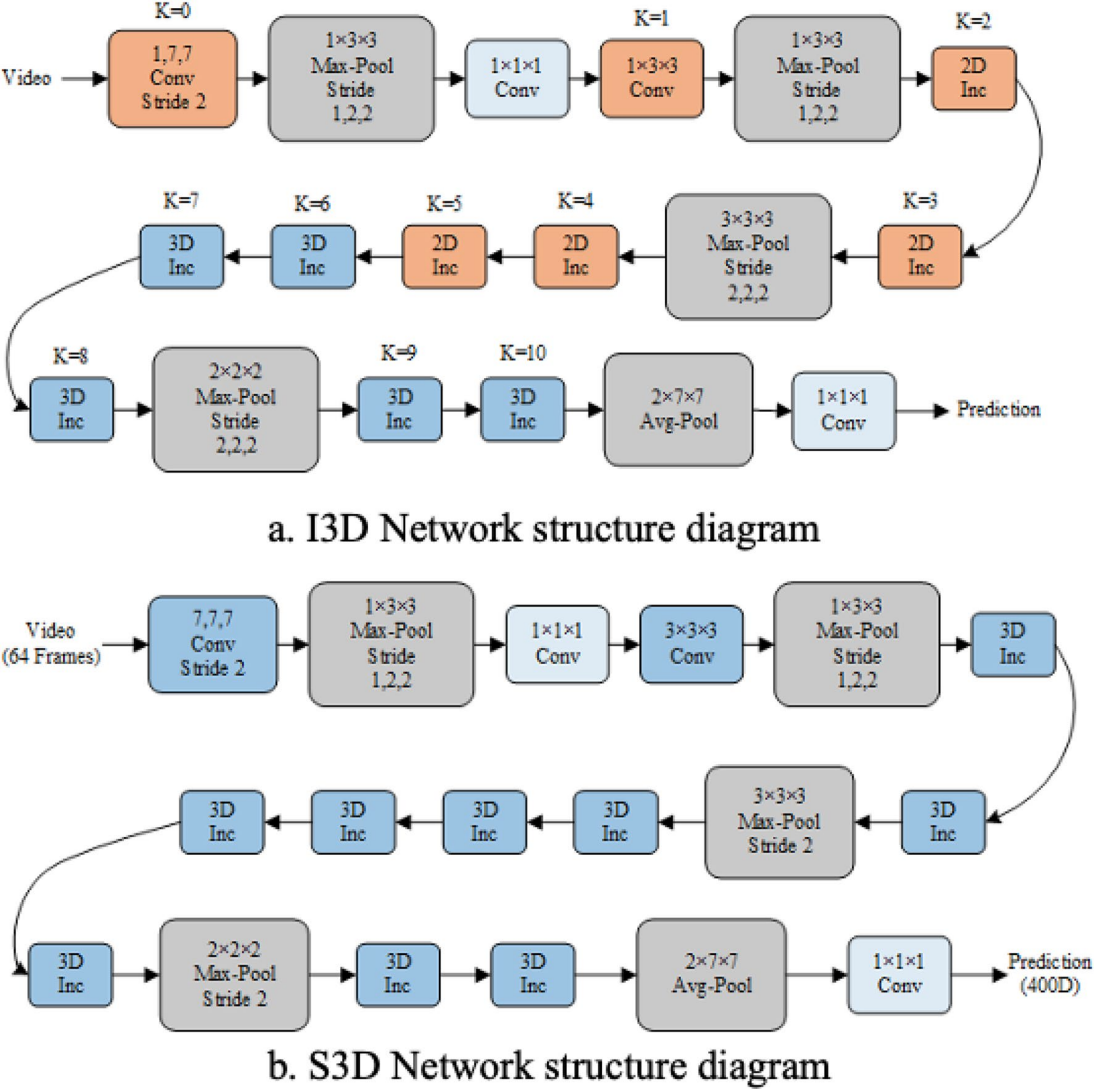
I3D and S3D network structure diagram.

The commonly used training datasets for 3D convolutional models, such as UCF101, Kinetics, and Something-Something^38^, exhibit a wide range of action categories and contain a considerable number of sub-videos for each action category. When all videos are processed into continuous frame sequences, these datasets produce a large number of frames. The transformation from 2D to 3D convolution such as I3D and S3D, is illustrated in Fig. 4. This process involves 2D convolution, 3D convolution, and feature extraction. This approach enables the network to capture both spatial and temporal information from the video, which is critical for accurate human behavior recognition. However, in terms of model calculation complexity, this process can cause great increase in the number of parameters, which lead to increased GPU load, decreased model training efficiency, and hinder further network optimization.

The computations for 2D and 3D convolutions are as follows:

1 $$\begin{aligned} V_{i j}^{x, y}= & {} \tanh \left( b_{i j}+\sum _{m} \sum _{p=0}^{p_{i}-1} \sum _{q=0}^{q_{i}-1} \omega _{i j m}^{p q} v_{(i-1) m}^{(x+p)(y+q)}\right) \end{aligned}$$

2 $$\begin{aligned} V_{i j}^{x, y, z}= & {} \tanh \left( b_{i j}+\sum _{m} \sum _{p=0}^{p_{i}-1} \sum _{q=0}^{q_{i}-1} \sum _{r=0}^{r_{i}-1} \omega _{i j m}^{p q r} v_{(i-1) m}^{(x+p)(y+q)(z+r)}\right) \end{aligned}$$

**Figure 5:**
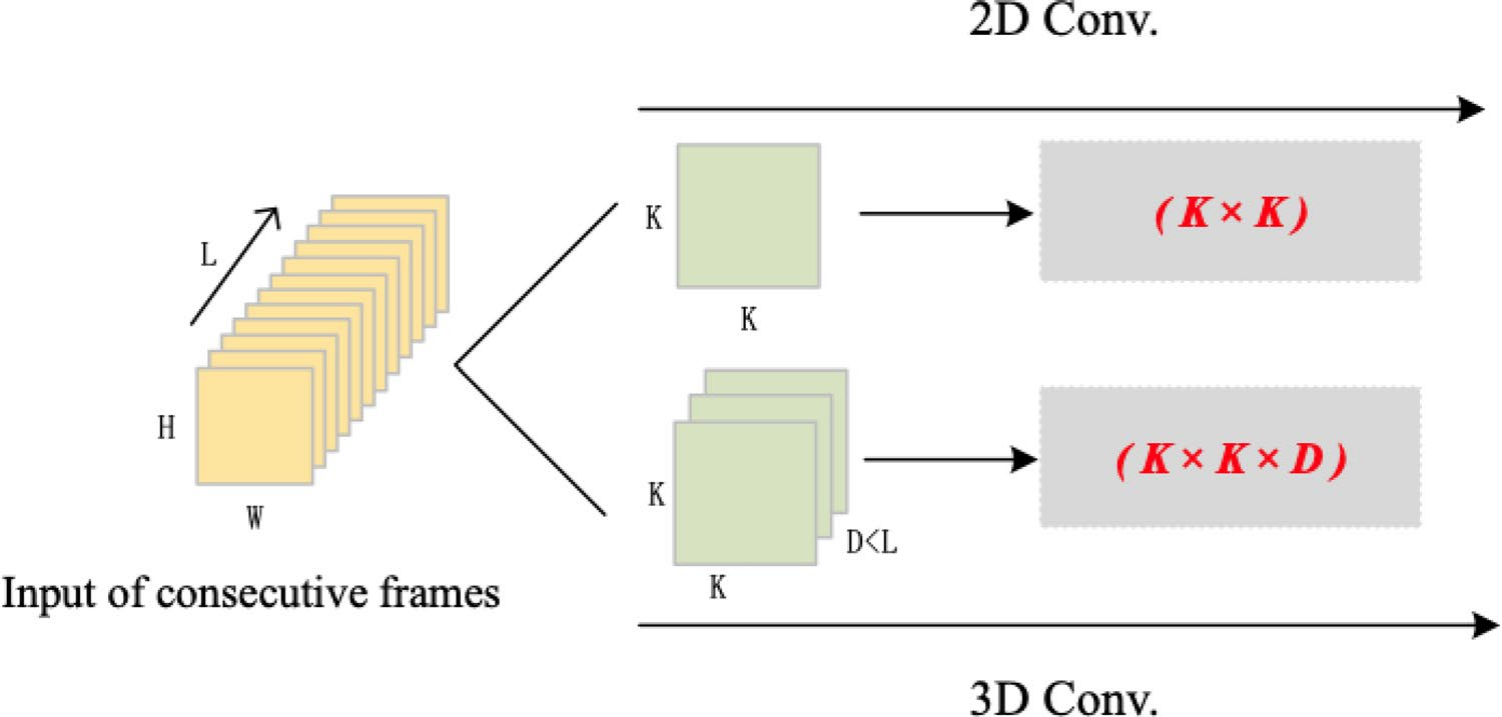
Comparison of 2D and 3D convolution parameters.

The 3D convolution operation is applied to each frame of the input continuous video individually, followed by the addition of convolved results with bias terms and application of the hyperbolic tangent (tanh) operation. The number of parameters associated with this operation is solely dependent on the size of the convolution kernel. The number of parameters of 2D and 3D convolution is illustrated in Fig. 5. This process involves a comparison of the parameter count between 2D and 3D convolutional networks when processing a video with dimensions $(H\times W \times L)$. It can be seen that the increase in the dimensions of the convolution kernel result in an increase in the number of calculation parameters and a significant increase in computational complexity.

### ATC module embedding method

**Figure 6:**
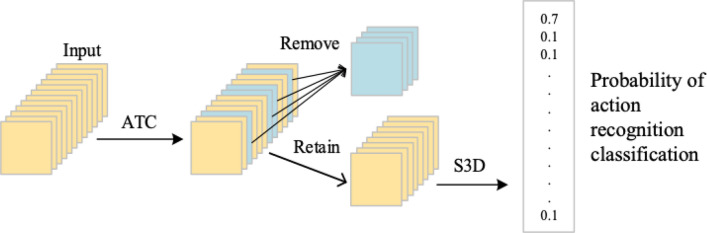
ATC module and network integration process.

The proposed ATC module is designed as a sub-network module, akin to the SENet^39^ and Inception^1,40,41^, that can be easily incorporated into existing deep learning networks without the need for any modifications. As a result, ATC is a plug-and-play module that can be readily integrated into any network architecture. Figure 6 demonstrates the integration of the ATC module with S3D, which serves as the base network in this study.

First, the video data formed by connecting image frames is input into the ATC module. Subsequently, the identified redundant frames are removed. The remaining video frames are sequentially concatenated to obtain new video data. Then, these video data serve as the compressed dataset input into the recognition network. Lastly, the Softmax layer of the network outputs the probability of the behavior class. The model’s overall performance is evaluated by analyzing the probability values and calculating Top-1 and Top-5 scores. Moreover, the S3D algorithm can be replaced with any other algorithm used for human behavior recognition. Placing the ATC module before the deep neural network allows for the compression of video frames prior to network input. This can lead to a reduction in computation and a faster training speed for the network.

**Figure 7:**
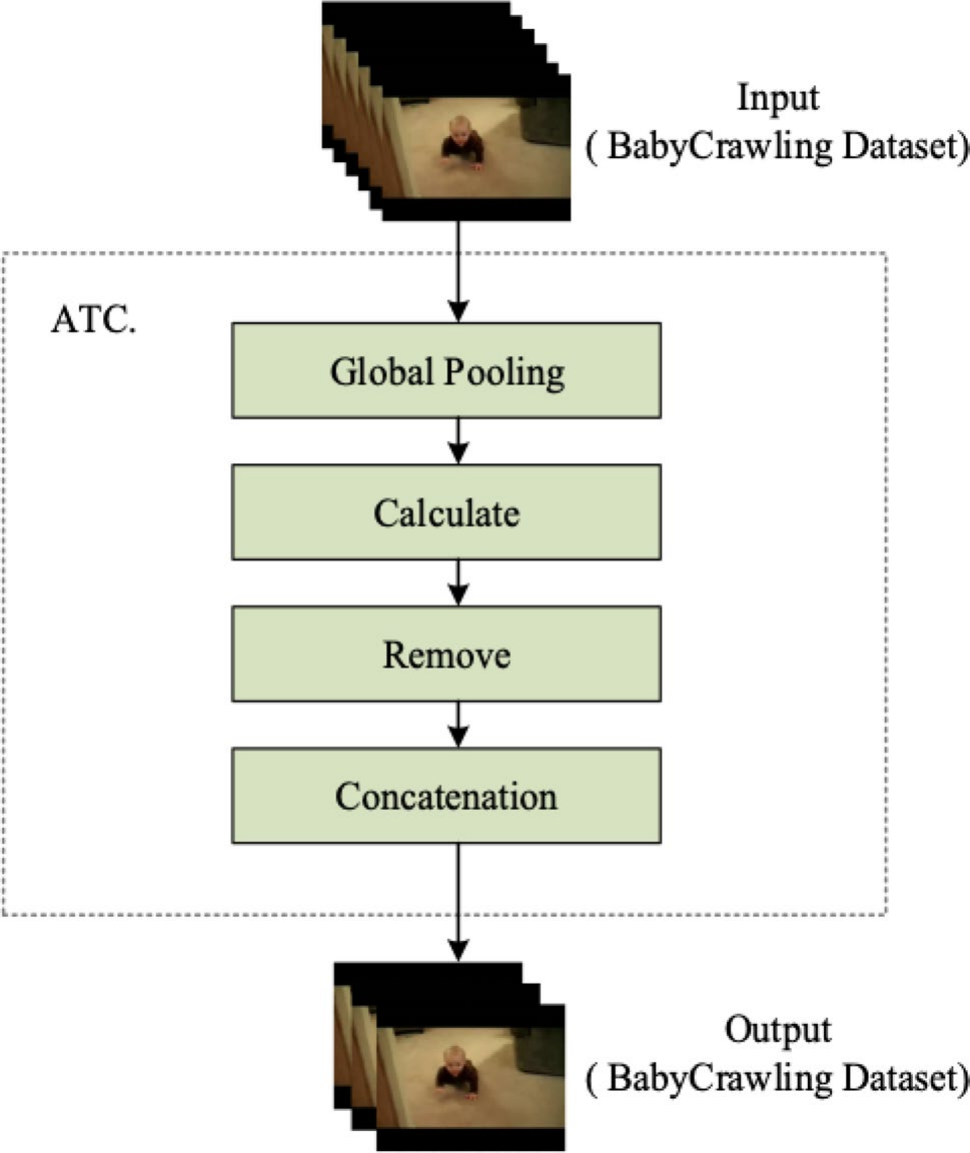
ATC module calculation flow chart.

The computational process of the ATC module is illustrated in Fig. 7, using the BabyCrawling dataset as an example. The module consists of Global Pooling, Calculation, Removal and Concatenation parts. First, the input continuous video frames undergo global pooling, transforming the dimensions from $(H\times W \times T)$ to a one-dimensional vector of $(1\times 1 \times T)$. T represents the number of frames in each input continuous video, while H and W are the height and width of the image, respectively. The global pooling operation is computed as follows:

3 $$\begin{aligned} Z_{c}=\frac{1}{W \times H} \sum _{i=1}^{W} \sum _{j=1}^{H} u_{c}(i, j) \end{aligned}$$

The second part of the ATC module involves the calculation of similarity for each element in the one-dimensional vector, followed by setting a threshold value. Two forms of similarity calculation are available: the ratio and the difference. In this study, we use the difference value to calculate the similarity. If the calculated ratio P is greater than the threshold value, the frame is deemed highly similar, i.e., redundant, and removed. On the other hand, if the calculated difference D is less than the threshold value, the frame is also deemed redundant and deleted. To prevent negative values in the vector, we use the absolute value operation on the difference value. The proportion and deviation is calculated as follow:

4 $$\begin{aligned} \text{P}= & {} \frac{x_{i}}{ \text{ Norm } _{i}} \end{aligned}$$

5 $$\begin{aligned} \text{D}= & {} \left| N_{\text{ norm } _{i}}-x_{i}\right| \end{aligned}$$

${x_{i}}$ represents the corresponding element value obtained after each video frame undergoes temporal dimension pooling. $N_{\text{ norm } _{i}}$ denotes the mean of the elements. The calculated P and D values are used to determine the similarity of video frames. In the final step of the ATC module, the redundant frames are removed, and the remaining frames are integrated in original order and compressed to create a new dataset. The time axis of the new dataset is compressed, which eliminates the need for the convolutional network to process all the video frames, thereby reducing the time complexity of the original dataset. This compressed dataset is then passed to the next stage of the network for further processing and classification.

**Figure 8:**
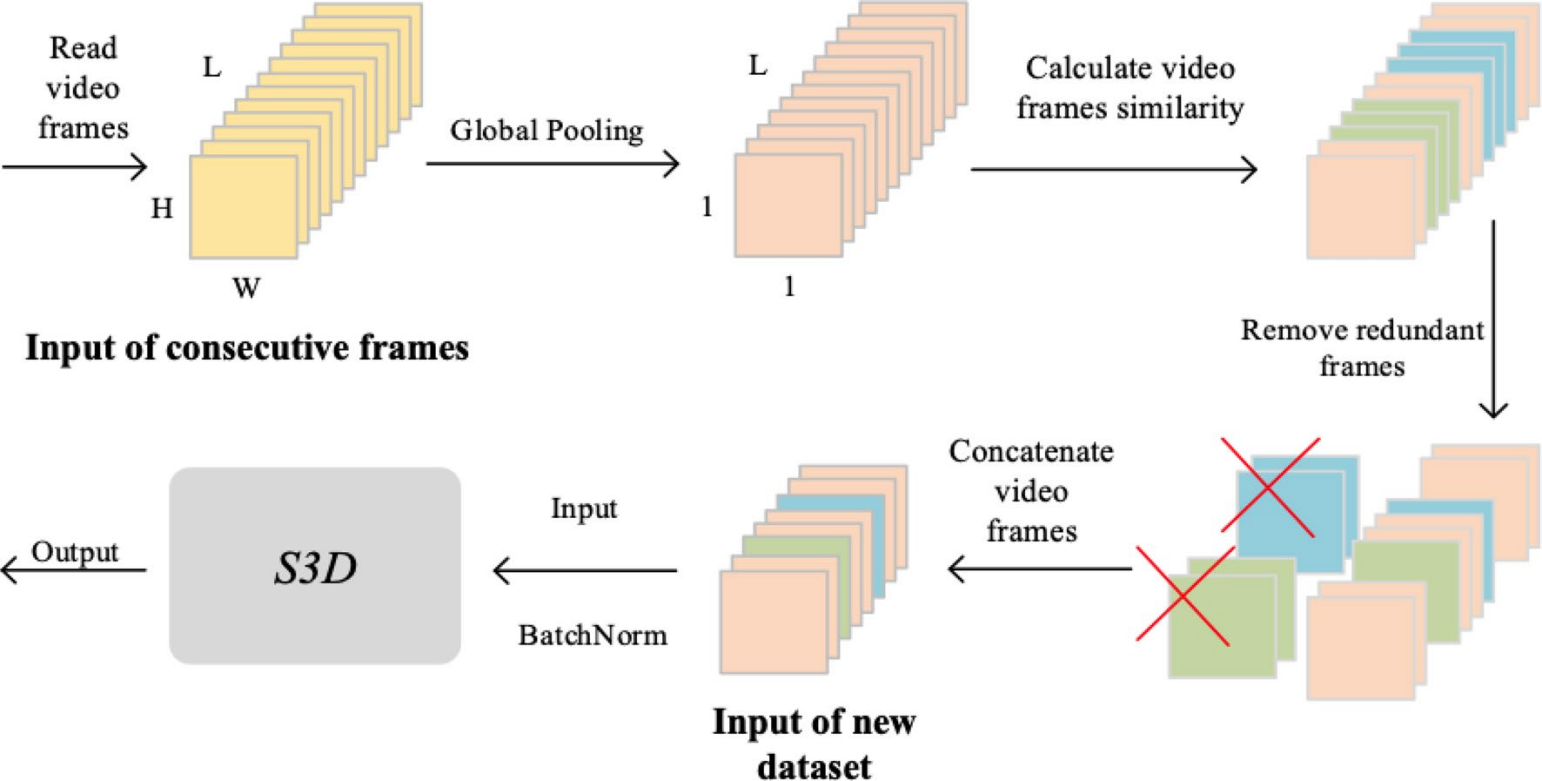
ATC module working visualization flow diagram.

Initially, the ATC module reads the entire sequence of continuous video frames. Subsequently, the module performs global pooling and calculates the similarity of the frames. As shown in Fig. 8, the green and blue regions represent the frames with high similarity, while the redundant frames are identified by comparing threshold values and are removed. The remaining contiguous frames are concatenated with the first and last frames, forming the output of the ATC module. Finally, the output is normalized using batch normalization and used as input to the S3D deep convolutional network for training and generating the desired output.

The batch normalization of mini batch mean, mini batch variance, normalize, and scale and shift are shown as follow, respectively^42–45^:

6 $$\begin{aligned} \mu _{B}= & {} \frac{1}{m} \sum _{i=1}^{m} x_{i} \end{aligned}$$

7 $$\begin{aligned} \sigma _{B}^{2}= & {} \frac{1}{m} \sum _{i=1}^{m}\left( x_{i}-\mu _{B}\right) ^{2} \end{aligned}$$

8 $$\begin{aligned} \widehat{x}_{l}= & {} \frac{x_{i}-\mu _{B}}{\sqrt{\sigma _{B}^{2}+\epsilon }} \end{aligned}$$

9 $$\begin{aligned} y_{i}= & {} \gamma \widehat{x}_{l}+\beta =B N_{\gamma , \beta }\left( X_{I}\right) \end{aligned}$$

where $\gamma$, $\beta$ are the learnable reconstruction parameter, $\mu _{B}$, $\sigma _{B}^{2}$ are the mean and variance respectively, $\hat{X_{l}}$ is the normalized result.

## Experiments and results

This study utilized two widely recognized human action recognition datasets, namely UCF101^9^ and Kinetics^10–13^, both of which encompass a substantial number of action categories. UCF101 dataset consists of 13,320 videos and 101 distinct action categories, encompassing a variety of sports-related actions sourced from BBC/ESPN, other broadcast TV channels, and YouTube. On the other hand, Kinetics dataset, provided by Google’s DeepMind team, was used for the Trimmed Action Recognition competition, and comprises of 700 action categories and approximately 600 video clips from various YouTube videos. Each of these clips is roughly 10 seconds long and involves various interactions between people and objects, such as playing musical instruments, interactions between people, handshakes and hugs, and physical activities and sports. These clips are further classified into person-object, person-person, and person-motion categories.

The experimental hardware setup consisted of a single NVIDIA GeForce GTX 2080Ti GPU with a VRAM capacity of 18GB, which was utilized for all deep learning computations. The PyTorch deep learning framework was employed to facilitate the development of the deep learning environment.

For the model training process, mini-batch stochastic gradient descent (SGD) was employed with a batch size of 32, momentum of 0.9, and weight decay of 1$e^{-4}$. The initial learning rate was multiplied by $(1-\frac{iter}{max\_iter})^{power}$, with a power of 0.9 for each iteration. The initial learning rate was set to 2.5$e^{-2}$.

The aim of first experiment was to evaluate the performance and efficacy of the C3D network integrated into the ATC module. The initial step involved training the C3D network directly, and the obtained weights were saved for further testing on the UCF101 test dataset. The experimental results comprised of Top-1, Top-5 accuracy and the testing time. Subsequently, the ATC module was added to the C3D network for identical tests, with the pre-trained parameters of Sports-1M being used as the initial parameters for both training sessions. The input dimensions for both sessions were $16\times 112\times 112$ RGB continuous video frames. Table 1 illustrates the comparison of the experimental results. The findings indicated that the ATC embedding reduced the model training and testing time by 24.35%, while maintaining accuracy.

**Table 1:** C3D model performance comparison.

Method	Input	Pre-training	TOP-1(%)	TOP-5(%)	Temporal Footprint
C3D	RGB	Sports-1M	76.8	82.5	35.47s
**C3D+ATC**	RGB	Sports-1M	76.5	82.3	26.83s

The objective of second experiment is to exhibit the practicality and efficacy of the ATC module. First, the S3D is chosen as the base network model for this experiment, and its performance is compared with the model’s performance after incorporating the ATC module. The Kinetics dataset was employed for the test. The experimental results indicate that the model testing speed increased by 52.09% compared to I3D and 38.86% compared to S3D, after adding the ATC module, see Table 2. Second, this experiment also compares the performance of the ATC module with the I3D network. The S3D network is a refined and optimized version of the I3D network, which substitutes the convolution in the network with separable operation in the temporal and spatial domains. To fully investigate the effect of utilizing spatio-temporal information, it is necessory to conduct a comprehensive assessment of all three networks. RGB is used as the input, and ImageNet^46,47^ pre-trained parameters are used as the initial parameters. The continuous video frame size for the input is set to $64\times 224\times 224$.

**Table 2:** S3D model performance comparison.

Method	Input	Pre-training	TOP-1(%)	TOP-5(%)	Temporal footprint
I3D	RGB	ImageNet	71.1	89.3	8.55s
S3D	RGB	ImageNet	72.2	90.6	6.87s
**S3D+ATC**	RGB	ImageNet	72.3	90.6	4.20s

Furthermore, this study includes a comparison of the overall performance of the network with others that used for human behavior recognition. The comparison is conducted using Kinetics dataset. The experimental results demonstrate that the S3D network embedded with the ATC module achieves a speedup of up to 38.86%, see Table 3. The comparison is not only limited to RGB input video frames, but also includes optical flow and a combination of both as input.

**Table 3:** Network performance comparison with Kinetics dataset.

Method	Input	Backbone	TOP-1(%)	TOP-5(%)	Temporal footprint
NL-I3D	RGB	ResNet-101	77.7	93.3	8.73s
I3D	RGB	Inception	71.1	89.3	8.55s
R(2+1)D	RGB	ResNet-34	74.3	91.4	7.03s
S3D	RGB	Inception	72.2	90.6	6.87s
**S3D+ATC**	RGB	ImageNet	72.3	90.6	4.20s

During the model computation, the number of video frames in the input data is directly proportional to the computational cost. For instance, fewer video frames result in lower computational costs. After processing the actions Kayaking and Baby Crawling from the UCF-101 dataset, as well as playing basketball and Spring Board$\_$diving from the Kinetics dataset through the ATC module, the number of video frames reduced by 26.20$\%$, 20.08$\%$, 13.87$\%$, and 19.00$\%$, respectively, as shown in Table 4. The YOLOv7^48^ algorithm is a fast and powerful network architecture that achieves high detection accuracy. This study compared the detection time of networks using the YOLOv7 model with and without the embedded ATC module. The test data consisted of video data of actions including Kayaking and Baby Crawling from the UCF-101 dataset, as well as playing basketball and Spring Board$\_$diving from the Kinetics dataset. The results demonstrate that the detection time of networks with the embedded ATC module is reduced by 21.54$\%$ and 21.35$\%$, 14.48$\%$ and 20.02$\%$ compared to the original networks, as shown in Table 5.

**Table 4:** Comparison of video frame counts for UCF-101 dataset after processing with the ATC module.

Category	Video frame counts without ATC	Video frame counts with ATC	Reduced (%)
Kayaking	28039	20694	26.20
Baby Crawling	21740	17374	20.08
Playing Basketball	249601	214971	13.87
Spring Board Diving	64733	52431	19.00

**Table 5:** Performance comparison of YOLOv7 and the ATC module on the UCF-101 and Kinetics datasets.

Category	YOLOv7 processing time (s)	YOLOv7+ATC processing time (s)	Reduced (%)
Kayaking	439.16	344.57	21.54
Baby crawling	333.19	206.06	21.35
Playing basketball	3586.89	3067.36	14.48
Spring board diving	926.57	769.91	20.02

**Figure 9:**
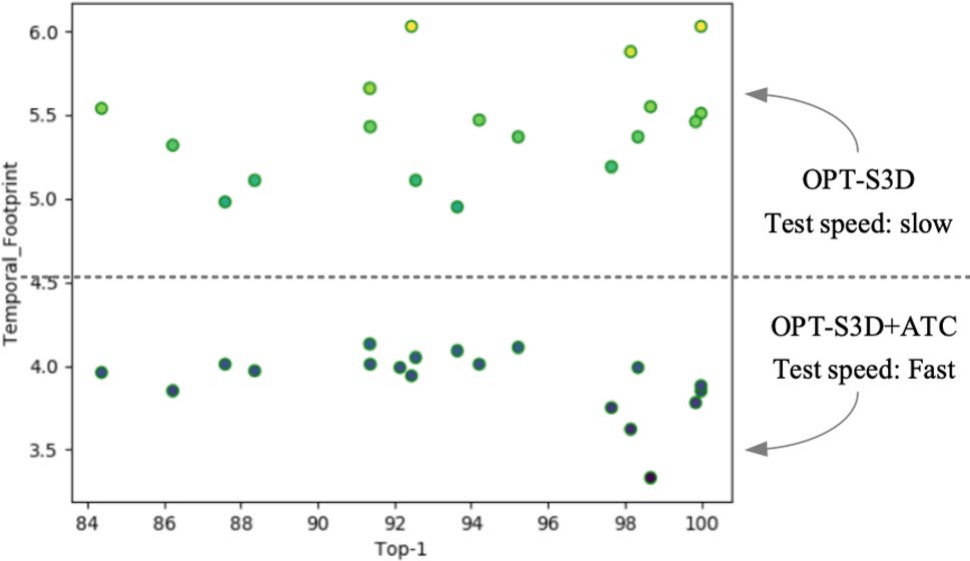
Comparison of detection speed and accuracy between the S3D network with and without the ATC module.

Lastly, 20 types of behaviors were randomly selected from the UCF101 dataset, and a comparison was made between the original model and the model embedded with the ATC module, see Fig. 9. It can be seen that embedding ATC module in models can significantly increase the efficiency for behavior recognition. This indicates the effectiveness of the ATC module in improving the model’s performance.

## Conclusion

To reduce computational costs at the data source, this paper proposes the video compression ATC module. ATC is a flexible plug-and-play module capable of compressing data by removing redundant frames during network training. It can be widely applied to tasks involving human behavior recognition using video data. Experimental results demonstrate that the ATC module can reduce time complexity with negligible loss of accuracy. Moreover, as a seamlessly integrable module, it offers high flexibility. However, the ATC module has limited impact on improving model accuracy. In future research, for recognition tasks with graph-based data as network inputs, such as facial expression recognition (Face2nodes) and action recognition on skeleton-based data, we will improve the ATC module based on graph similarity. In summary, the ATC module exhibits strong scalability as a general method, with many potential functionalities awaiting exploration.

## Data Availability

The datasets analysed during the current study available from the corresponding author on reasonable request.
